# Is valve-sparing aortic root replacement better than total aortic root replacement? An overview of reviews

**DOI:** 10.3389/fcvm.2023.1115290

**Published:** 2023-04-18

**Authors:** Wei Wang, Xuezhou Zhang, Yong Shi, Siqi Xu, Teng Shi, Xiaotian Han, Tianxiang Gu, Enyi Shi

**Affiliations:** Department of Cardiac Surgery, First Affiliated Hospital, China Medical University, Shenyang, China

**Keywords:** total aortic root replacement, valve-sparing aortic root replacement, aortic surgery, overview of reviews, systematic reviews

## Abstract

**Background:**

Total aortic root replacement (TRR) is certainly beneficial for aortic root disease, but does it still have an advantageous prognosis for patients compared to valve-sparing aortic root replacement (VSRR)? An overview of reviews was conducted to assess each of their clinical efficacy/effectiveness.

**Review methods:**

Systematic reviews (SRs)/Meta-analyses comparing the prognosis of TRR and VSRR in aortic root surgery were collected from 4 databases, all searched from the time of database creation to October 2022. Two evaluators independently screened the literature, extracted information and applied the Preferred Reporting Items for Systematic Reviews and Meta-Analyses (PRISMA) statement, A Measurement Tool to Assess Systematic Reviews 2 (AMSTAR 2) tool, Grading of Recommendations, Assessment, Development and Evaluations (GRADE), and Risk of Bias in Systematic Reviews (ROBIS) to evaluate the quality of reporting, methodological quality, risk of bias, and level of evidence of the included studies.

**Main results:**

A total of 9 SRs/Meta-analyses were ultimately included. In terms of the reporting quality of the included studies, PRISMA scores ranged from 14 to 22.5, with issues mainly in reporting bias assessment, risk of study bias, credibility of evidence, protocol and registration, and funding sources. The methodological quality of the included SRs/Meta-analyses was generally low, with key items 2, 7, and 13 having major flaws and non-key items 10, 12, and 16. In terms of risk of bias assessment, the overall assessment of the included 9 studies was high-risk. The quality of the evidence was rated as low to very low quality for the three outcome indicators selected for the GRADE quality of evidence rating: early (within 30 days postoperatively or during hospitalization) mortality, late mortality, and valve reintervention rate.

**Conclusions:**

VSRR has many benefits including reduced early and late mortality after aortic root surgery and reduced rates of valve-related adverse events, but the methodological quality of the relevant studies is low, and there is a lack of high-quality evidence to support this.

**Systematic Review Registration:**

https://www.PROSPERO, identifier: CRD42022381330.

## 1. Introduction

TRR using a composite mechanical valve, as proposed by Bentall and De Bono in 1968 (1), has been a boon to many patients requiring surgery for aortic root disease. For more than 50 years, it has long been considered the “gold procedure” for aortic root disease, particularly type A aortic dissection and Marfan syndrome, because of its excellent early and late postoperative results (2, 3). However, the implementation of a mechanical prosthesis exposes patients to a cumulative risk of lifelong anticoagulation, hemodynamic restrictions, and an increased risk of thromboembolism. Even though bio-prosthesis implantation can minimize these risks, re-intervention would be an undesired result of bio-prosthesis degeneration (4–6).

Although TRR is the most common procedure performed during surgery for aortic root disease, the optimal management of the aortic valve at the time of root surgery remains highly controversial. The benefit of preserving the native aortic valve, particularly in some young patients with good aortic valve pathology, has been remarkable. This controversy has become more intense since the introduction of the reimplantation technique by David in 1992 (7) and the remodeling technique by Yacoub in 1983 (8). The superior early outcome, lower late cardiac-related mortality, and valve-related complications of VSRR have led to a strong preference (6, 9, 10). Because VSRR is so challenging, most studies have come from specialist cardiac centers. Some argue that the more technically demanding VSRR has a proportionally increased complication and mortality rate, both intraoperatively and postoperatively. In this way, the prognosis of patients who undergo VSRR is not necessarily better than those who opt for a composite mechanical or biological valve for TRR (11).

A large number of studies exist that have explored the early and late mortality and complications of TRR and VSRR, and several SRs/Meta-analyses have been published based on this. However, there is considerable heterogeneity in the original studies included in the various SRs/Meta-analyses in terms of year of publication, sample size, interventions/controls, and outcome indicators. In particular, the inconsistency of postoperative complication rates across different research has largely limited the application and dissemination of evidence-based evidence in clinical practice. Overview of reviews (Overviews) is a comprehensive approach to collecting systematic reviews on the etiology diagnosis, treatment, and prognosis of the same disease or health problem and conducting a comprehensive study (12). And this article aims to analyze the current published SRs/Meta-analyses on the prognosis of TRR compared to VSRR and provide a basis for clinical selection.

## 2. Materials and methods

The protocol for this overview was registered on PROSPERO (CRD42022381330) and is accessible on the PROSPERO website (https://www.crd.york.ac.uk/prospero/). The reporting of this overview of reviews adheres to the PRISMA 2020 criteria (13).

### 2.1. Search and study selection

PubMed, Embase, Web of science, and China National Knowledge Infrastructure (CNKI) databases were searched to collect SRs/Meta-analyses comparing survival, mortality, complications, and reoperation rates after VSRR versus TRR, all searched from the time of database creation to October 2022. In addition, references to the included literature were retrospectively included to supplement access to relevant literature. Searches were conducted using a combination of subject terms and free words. Terms include aortic valve-sparing, aortic valve preservation, aortic valve repair, aortic valve-sparing, VSRR, David procedure, remodeling, Yacoub, reimplantation, Bentall procedure, composite valve graft, valved conduit, CVG, total root replacement, aortic valve replacement, systematic review, meta-analysis, etc. The specific search strategy for PubMed, for example, is shown in Table 1.

**Table 1:** The search strategy using pubmed as an example.

SET	QUERY
#1	aortic valve-preserving [Title/Abstract]
#2	aortic valve preservation [Title/Abstract]
#3	aortic valve repair [Title/Abstract]
#4	aortic valve-sparing [Title/Abstract]
#5	VSRR [Title/Abstract]
#6	David procedure [Title/Abstract]
#7	remoulding [Title/Abstract]
#8	remodeling [Title/Abstract]
#9	reimplantation [Title/Abstract]
#10	Yacoub [Title/Abstract]
#11	#1 OR #2 OR #3 OR #4 OR #5 OR #6 OR #7 OR #8 OR #9 OR #10
#12	Bentall procedure [Title/Abstract]
#13	composite valve graft [Title/Abstract]
#14	valved conduit [Title/Abstract]
#15	CVG [Title/Abstract]
#16	total root replacement [Title/Abstract]
#17	aortic valve replacement [Title/Abstract]
#18	#12 OR #13 OR #14 OR #15 OR #16 OR #17
#19	#11 AND #18
#20	“Meta-Analysis as Topic” [Mesh]OR“Meta-Analysis”[Publication Type]
#21	meta analysis[Title/Abstract] OR meta analyses[Title/Abstract] OR meta-analysis[Title/Abstract] OR meta-analyses[Title/Abstract] OR metaanalysis[Title/Abstract] OR metanalysis[Title/Abstract] OR met-analysis[Title/Abstract]OR meta analyses[Title/Abstract]OR metanalyses[Title/Abstract]OR met-analyses[Title/Abstract] OR data pooling[Title/Abstract]OR data poolings[Title/Abstract]
#22	#20 OR #21
#23	systematic review[Title/Abstract] OR systematic reviews[Title/Abstract]
#24	#22 OR #23
#25	#19 AND #24

### 2.2. Inclusion and exclusion criteria

**SRs/Meta-analyses were included if:**

(i) Review of studies on the clinical outcomes of TRR and VSRR.
(ii) Research into aortic root diseases, including but not limited to aortic dissection and Marfan syndrome.
(iii) Primary outcome indicators include early mortality (within 30 days of surgery or during hospitalization), mortality during follow-up, reoperation rates, thromboembolic events, endocarditis, and bleeding associated with aortic root and aortic valve lesions.

**SRs/Meta-analyses were excluded if:**

(i) Reviews, conference abstracts, case reports, and letters.
(ii) Duplicate publications or overlapping studies included.
(iii) Literature for which data could not be extracted or full text was not available.
(iv) Currently incomplete SRs/Meta-analyses.

### 2.3. Literature screening and data extraction

Two evaluators independently screened the literature, extracted information, and cross-checked it, consulting a third person to assist with any disagreements and contacting the authors to supplement any missing information where possible. The literature was screened by first reading the title and abstract and then, after excluding any irrelevant literature, further reading the full text to determine final inclusion. If multiple SRs/Meta-analyses existed for the same group of researchers, those with a relatively recent year of publication and containing more complete studies were selected for inclusion. Data extraction included the following: first author and year of publication, number of included studies, sample size, interventions, risk of bias assessment tools used, outcome indicators, PRISMA score (14) results, AMSTAR 2 (15) evaluation results and funding sources.

### 2.4. Assessment of included reviews

All of the evaluation methods were assessed independently by two researchers and then summarized. Any inconsistencies were resolved by consensus or by third-author adjudication.

#### 2.4.1. Reporting quality

The PRISMA Statement (14) evaluates the quality of the reports included in the study using a total of 27 items, with each item being scored 1 for complete reporting, 0.5 for partial reporting, and 0 for no reporting, out of a total of 27 points, with a score of <15 being considered a relatively serious information deficiency in the systematic evaluation report, a score of 15–21 being considered some deficiency in the report, and a score of 21 or more being considered a relatively complete report. The PRISMA statement indicates that a report with a completeness level of <50% for each item is considered to be deficient.

#### 2.4.2. Methodological quality

The AMSTAR 2 (15) scale was used to evaluate the methodological quality of the included studies. The scale contains 16 items, of which items 2, 4, 7, 9, 11, 13, and 15 are key items, and the results are classified into three levels: satisfied, partially satisfied, and not satisfied. AMSTAR 2 scores of satisfied and partially satisfied ≥70% are considered to be a complete report of the items.

#### 2.4.3. Evidence quality

GRADE (16) was used to evaluate the quality of evidence for different outcome indicators of the included studies, with downgrading factors including study limitations, inconsistency of findings, non-directness or indirectness (uncertainty about whether it is direct evidence), imprecision (insufficient precision or wide confidence intervals), and publication bias. The quality of the evidence was graded into four categories: high, moderate, low, and very low.

#### 2.4.4. Risk of bias

The level of bias in each of the included SRs was assessed using the ROBIS tool (17), which helps to assess the level of bias in four domains: (1) eligibility criteria for each study; (2) study identification and selection; (3) data collection and study evaluation; and (4) overall synthesis and key findings. Within each domain, specific questions were used to determine the risk of bias, with bias rated as “low”, “high” or “uncertain”.

### 2.5. Data synthesis and application of software

Due to the heterogeneity between SRs, particularly between the TRR and VSRR groups, and the duplication of studies included in the individual RCTs, the selected SRs were analyzed descriptively only, rather than quantitatively synthesized. The data were summarized as percentages and frequencies for each of PRISMA, AMSTAR 2, GRADE, and ROBIS. The characteristics and results of each SR and these tools' results are presented in tables and figures using RStudio and Review Manager (RevMan).

## 3. Results

### 3.1. Literature selection and basic characteristics

**Figure 1:**
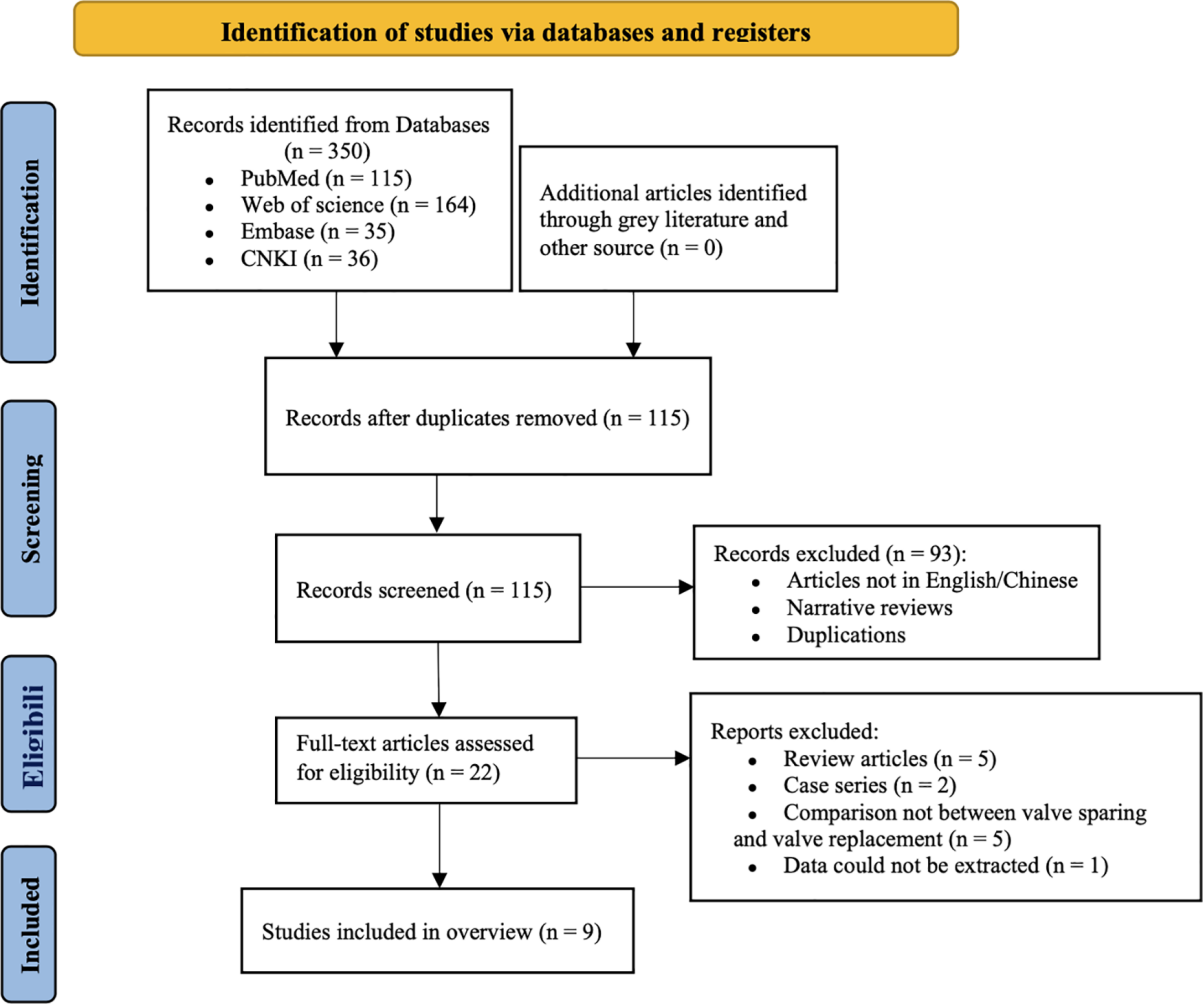
Flow diagram of the search and screening of the literature included in this study.

The initial literature search identified 350 potential SRs/Meta-analyses. Duplicate publications were removed by filtering (*n* = 235). After screening all titles and abstracts, 93 articles were excluded and the remaining 22 articles were retrieved for further review. After screening 22 full-text articles, 13 SRs were excluded and 9 SRs/Meta-analyses (18–26) were ultimately included. The literature screening process and results are shown in Figure 1. The basic characteristics of the included studies are shown in Table 2.

**Table 2:** Characteristics of the included systematic reviews and the score of PRISMA evaluation.

First author	Year	Intervention		No. of studies	Sample size	Included diseases or methods of operation	Outcomes
Soto	2021	VSRR	TRR	41	4,025	MFS and other connective tissue diseases	In-hospital and late mortality, stroke, bleeding, aortic insufficiency, endocarditis, thromboembolic events, arrhythmia, valve reintervention, freedom from valve reintervention
Burgstaller	2018	RAV	CVG	20	21,560	MFS	In-hospital deaths, mortality/survival during the follow-up period, reoperation related to aortic root and aortic valve diseases, reoperation rate because of bleeding, stroke rate, thromboembolism and pacemaker implantation during the hospital stay
Benedetto	2011	VSRR	TRR	11	13,850	MFS	Reintervention on the aortic valve, thromboembolic event, endocarditis
Elbatarny	2020	VSRR	CVG	26	6,218	Aortic root dilation	All-cause mortality, reoperation for bleeding, myocardial infarction, thromboembolism/stroke, reintervention, bleeding
Flynn	2017	VSRR	CVG	23	2,976	MFS	Endocarditis, thromboembolism, hemorrhagic complication and reoperation
Hu	2014	VSRR	TRR	7	690	MFS	Thromboembolism, endocarditis, long-term death, re-exploration, reoperation rates
Mosbahi	2018	RAV	CVG	27	3,058	Acute type A aortic dissection	In-hospital mortality, mortality/survival during the follow-up, and reoperation related to the AoR and/or aortic valve pathology during the follow-up, reoperation because of bleeding, incidence of stroke, thromboembolic events and incidence of permanent pacemaker implantation during hospital stay.
Salmasi	2019	VSRR	Bentall	34	7,313	Aortic root aneurysms	In-hospital or up to 30 days post-surgery death, incidence of complications and time spent in intensive care/hospital, survival at various intervals, rates of reintervention, echocardiographic parameters and functional class
Wu	2019	VSRR	Bentall	9	706	Acute type A aortic dissection	Early mortality, late mortality, re-exploration, thromboembolic/bleeding events, post-operative infective endocarditis and reintervention.
First author	Year	Quality assessment tool	Data-analysis method	Subgroup/sensitivity analysis/meta-regression	Publication bias	Score of PRISMA evaluation	
Soto	2021	Grading of Recommendations Assessment, Development and Evaluation (GRADE) criteria	Meta-analysis	No/No/No	No	22.5	
Burgstaller	2018	Scottish Intercollegiate Guidelines Network (SIGN) Methodology checklist	Meta-analysis	No/No/Yes	Yes	18	
Benedetto	2011	Not mentioned	Meta-analysis	Yes/No/Yes	No	15	
Elbatarny	2020	Grading of Recommendations Assessment, Development and Evaluation (GRADE) criteria	Meta-analysis	Yes/Yes/Yes	Yes	20	
Flynn	2017	Not mentioned	Meta-analysis	Yes/Yes/No	No	15	
Hu	2014	Newcastle–Ottawa scale (NOS)	Meta-analysis	No/No/No	No	14	
Mosbahi	2018	Scottish Intercollegiate Guidelines Network (SIGN) Methodology checklist	Meta-analysis	Yes/No/Yes	Possibly Yes	17.5	
Salmasi	2019	Newcastle–Ottawa scale (NOS)	Meta-analysis	Yes/No/Yes	No	20.5	
Wu	2019	Newcastle–Ottawa scale (NOS)	Meta-analysis	No/Yes/Yes	No	18.5	

RAV, reimplantation of the aortic valve; CVG, composite valve graft; TRR, total aortic root replacement; VSRR, valve sparing aortic root replacement.

### 3.2. Quality evaluation of the included studies

#### 3.2.1. Reporting quality

The PRISMA (14) scores for the included studies ranged from 14 to 22.5 (Table 2). Of these, 1 study (23) scored 14 (reported relatively serious information deficiencies) and 7 studies (19–22, 24–26) scored ≤21 (reported some deficiencies). The PRISMA statement (14) items for which more than half of the studies were rated as “not satisfied” included: assessment of reporting bias, risk of study bias, credibility of evidence, protocol and registration, and funding source. PRISMA statement (14) items for which more than half of the studies were evaluated as “partially satisfied” included: a structured summary, inclusion/exclusion criteria, information sources, search strategy, data extraction, data items, synthesis of methods, and synthesis of results.

#### 3.2.2. Methodological quality

**Figure 2:**
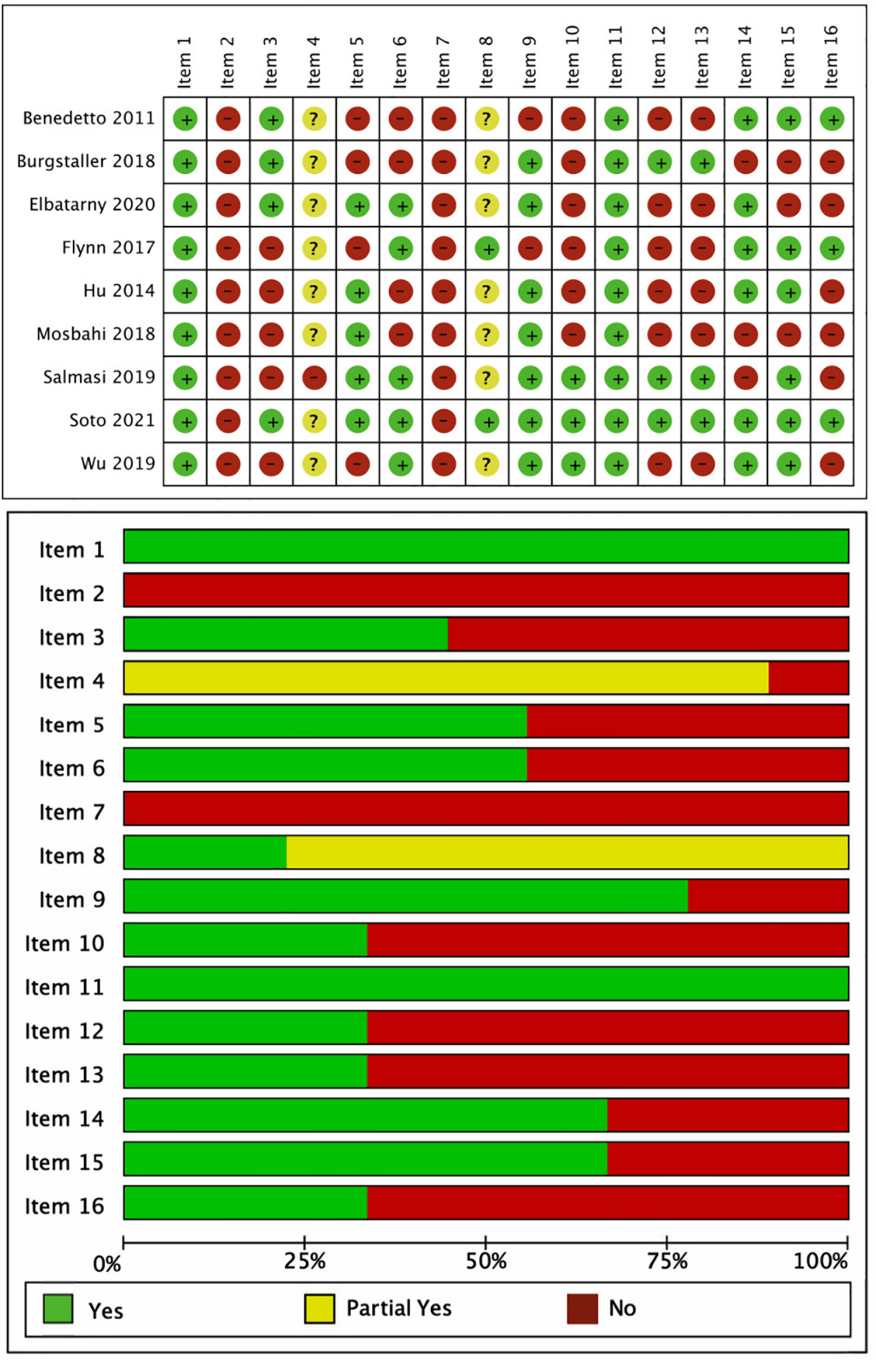
Results of the visualization quality evaluation of A Measurement Tool to Assess Systematic Reviews 2.

The results of the AMSTAR 2 (15) evaluation showed that the methodological quality of all studies was “very low” (Table 3). A total of 5 items with AMSTAR 2 scores of ≥70% satisfied and partially satisfied indicated high quality. Of the 7 critical items in the AMSTAR 2 (15) quality assessment, item 2, item 7, and item 13 had significant deficiencies; the non-critical items with significant deficiencies were item 10, item 12, and item 16. The results for each item in the AMSTAR 2 quality assessment are shown in Figure 2.

**Table 3:** Methodological quality assessment of included systematic reviews/meta-analyses by AMSTAR 2.

Item	First author and publication year									Compliance [*n* (%)]
	Soto 2021	Burgstaller 2018	Benedetto 2011	Elbatarny 2020	Flynn 2017	Hu 2014	Mosbahi 2018	Salmasi 2019	Wu 2019	
Item 1	Y	Y	Y	Y	Y	Y	Y	Y	Y	9 (100.00)
Item 2^a^	N	N	N	N	N	N	N	N	N	0 (0)
Item 3	Y	Y	Y	Y	N	N	N	N	N	4 (44.44)
Item 4^a^	PY	PY	PY	PY	PY	PY	PY	N	PY	8 (88.89)
Item 5	Y	N	N	Y	N	Y	Y	Y	N	5 (55.56)
Item 6	Y	N	N	Y	Y	N	N	Y	Y	5 (55.56)
Item 7^a^	N	N	N	N	N	N	N	N	N	0 (0)
Item 8	Y	PY	PY	PY	Y	PY	PY	PY	PY	9 (100.00)
Item 9^a^	Y	Y	N	Y	N	Y	Y	Y	Y	7 (77.78)
Item 10	Y	N	N	N	N	N	N	Y	Y	3 (33.33)
Item 11^a^	Y	Y	Y	Y	Y	Y	Y	Y	Y	9 (100.00)
Item 12	Y	Y	N	N	N	N	N	Y	N	3 (33.33)
Item 13^a^	Y	Y	N	N	N	N	N	Y	N	3 (33.33)
Item 14	Y	N	Y	Y	Y	Y	N	N	Y	6 (66.67)
Item 15^a^	Y	N	Y	N	Y	Y	N	Y	Y	6 (66.67)
Item 16	Y	N	Y	N	Y	N	N	N	N	3 (33.33)
Compliance [*n* (%)]	14 (87.50)	8 (50.00)	8 (50.00)	9 (56.25)	8 (50.00)	8 (50.00)	6 (37.50)	10 (62.50)	9 (56.25)	
Ranking of quality	Critical low	Critical low	Critical low	Critical low	Critical low	Critical low	Critical low	Critical low	Critical low	

^a^ Critical items of AMSTAR 2; AMSTAR 2: A Measurement Tool to Assess Systematic Reviews 2; Y, yes; PY, partial yes; N, no. Item 1: did the research questions and inclusion criteria for the review include the components of population, intervention, comparison, and outcome (PICO)? Item 2: did the report of the review contain an explicit statement that the review methods were established prior to the conduct of the review and did the report justify any significant deviations from the protocol? Item 3: did the review authors explain their selection of the study designs for inclusion in the review? Item 4: did the review authors use a comprehensive literature search strategy? Item 5: did the review authors perform study selection in duplicate? Item 6: did the review authors perform data extraction in duplicate? Item 7: did the review authors provide a list of excluded studies and justify the exclusions? Item 8: did the review authors describe the included studies in adequate detail? Item 9: did the review authors use a satisfactory technique for assessing the risk of bias (RoB) in individual studies that were included in the review? Item 10: did the review authors report on the sources of funding for the studies included in the review? Item 11: if meta-analysis was performed, did the review authors use appropriate methods for statistical combination of results? Item 12: if meta-analysis was performed, did the review authors assess the potential impact of RoB in individual studies on the results of the meta-analysis or other evidence synthesis? Item 13: did the review authors account for RoB in individual studies when interpreting/discussing the results of the review? Item 14: did the review authors provide a satisfactory explanation for, and discussion of, any heterogeneity observed in the results of the review? Item 15: if they performed quantitative synthesis, did the review authors carry out an adequate investigation of publication bias (small study bias) and discuss its likely impact on the results of the review? Item 16: did the review authors report any potential sources of conflicts of interest, including any funding they received for conducting the review?.

#### 3.2.3. Evidence quality

**Figure 3:**
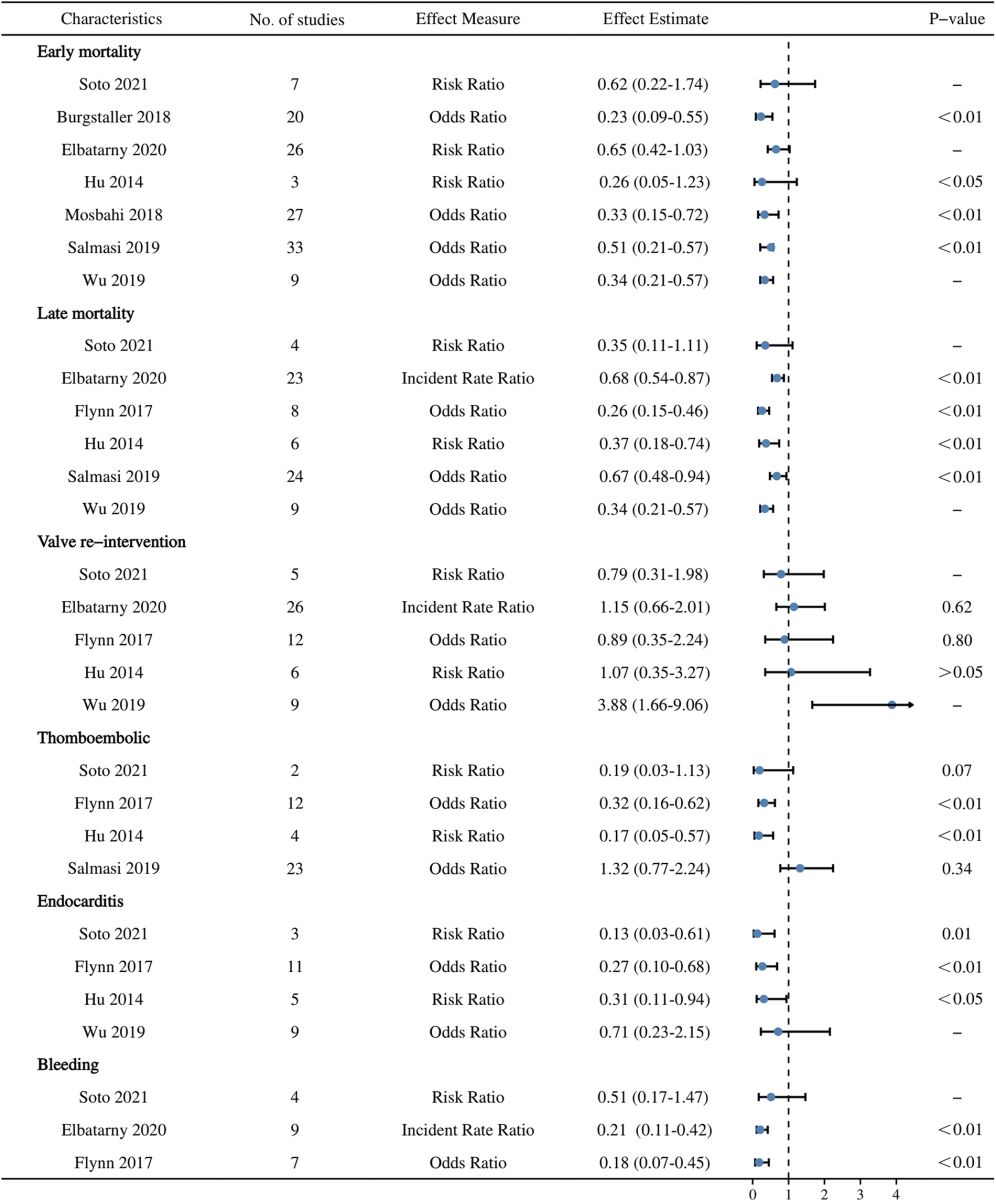
A summary of the 6 postoperative outcomes evaluated using Grading of Recommendations, Assessment, Development and Evaluations.

7 articles (18, 19, 21, 23–26) reporting early mortality were included, 3 (19, 21, 24) of which showed very low-quality evidence. 6 (18, 21–23, 25, 26) that reported late mortality, 5 (18, 21–23, 26) on valve re-intervention, and 3 (18, 21, 22) on bleeding were included, and they each showed the results of Elbatarny et al. (21) as very low-quality evidence. Except for the very low-quality evidence mentioned above, all were low-quality evidence. The main factors causing the downgrading of the quality of the evidence were Inconsistency, Indirectness, and Imprecision. The results are detailed in Table 4 and Figure 3.

**Table 4:** Main results assessed by the grading of recommendations, assessment, development and evaluations.

Main results	First author/Publication year	No. of studies	Risk of bias	Inconsistency	Indirectness	Imprecision	Publication bias	Quality of evidence
Early mortality	Soto 2021	7	0	0	0	0	0	Low
	Burgstaller 2018	20	−1	0	0	0	−1	Very low
	Elbatarny 2020	26	−1	0	0	0	−1	Very low
	Hu 2014	3	0	0	0	0	0	Low
	Mosbahi 2018	27	−1	0	0	0	−1	Very low
	Salmasi 2019	33	0	0	0	0	0	Low
	Wu 2019	9	0	0	0	0	0	Low
Late mortality	Soto 2021	4	0	0	0	0	0	Low
	Elbatarny 2020	23	−1	0	0	0	−1	Very low
	Flynn 2017	8	0	0	0	0	0	Low
	Hu 2014	6	0	0	0	0	0	Low
	Salmasi 2019	24	0	0	0	0	0	Low
	Wu 2019	9	0	0	0	0	0	Low
Valve re-intervention	Soto 2021	5	0	0	0	0	0	Low
	Elbatarny 2020	26	−1	0	0	0	−1	Very low
	Flynn 2017	12	0	0	0	0	0	Low
	Hu 2014	6	0	0	0	0	0	Low
	Wu 2019	9	0	0	0	0	0	Low
Thomboembolic	Soto 2021	2	0	0	0	0	0	Low
	Flynn 2017	12	0	0	0	0	0	Low
	Hu 2014	4	0	0	0	0	0	Low
	Salmasi 2019	23	0	0	0	0	0	Low
Endocarditis	Soto 2021	3	0	0	0	0	0	Low
	Flynn 2017	11	0	0	0	0	0	Low
	Hu 2014	5	0	0	0	0	0	Low
	Wu 2019	9	0	0	0	0	0	Low
Bleeding	Soto 2021	4	0	0	0	0	0	Low
	Elbatarny 2020	9	−1	0	0	0	−1	Very low
	Flynn 2017	7	0	0	0	0	0	Low

#### 3.2.4. Risk of bias

**Figure 4:**
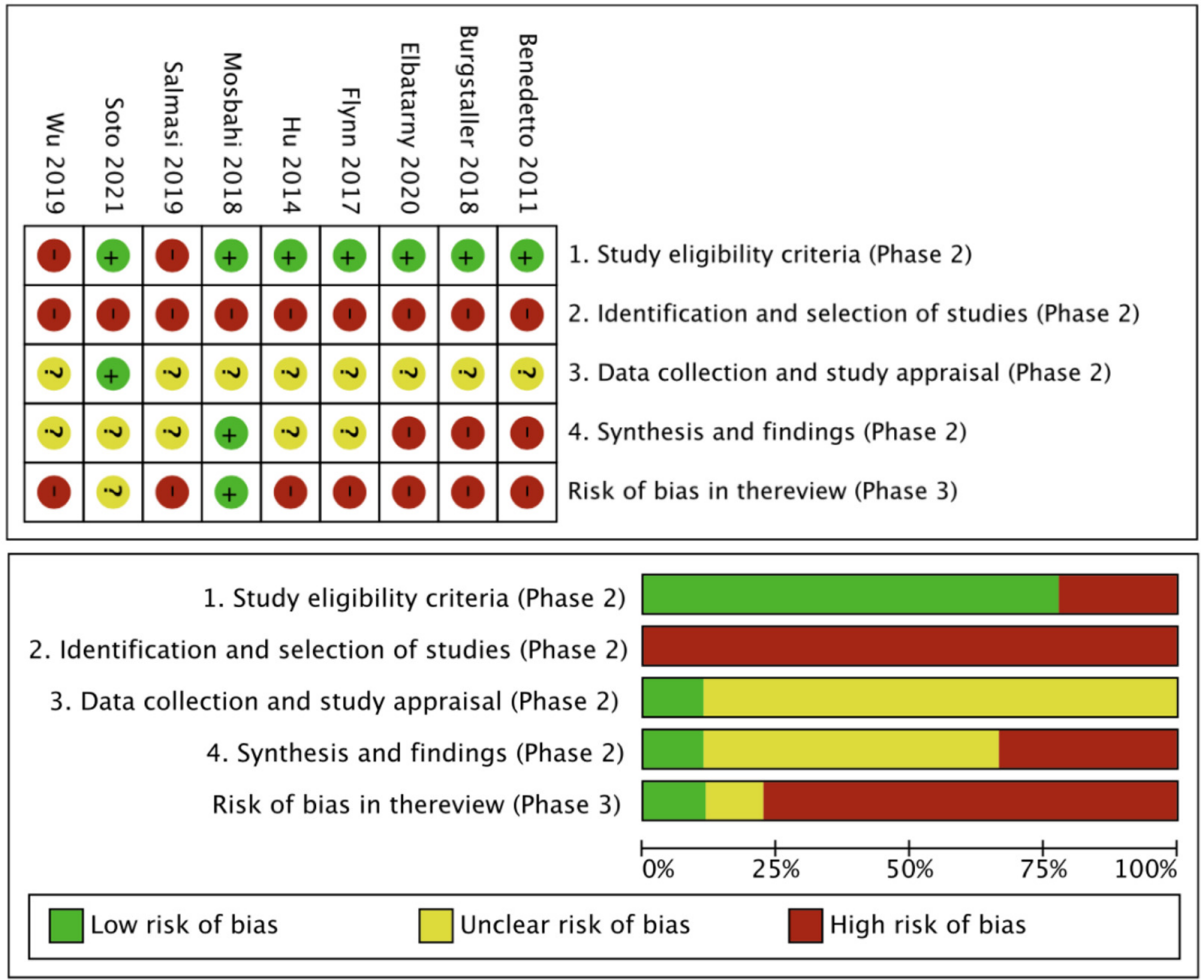
Visual analysis results of Risk of Bias in Systematic Reviews.

In this overview, we did not implement the first stage of ROBIS, which was used to determine whether the proposed question and the target question matched. Using Domain-1 to assess the inclusion and exclusion criteria for each study, we found that 77.8% (7/9) of the SRs/Meta-analyses (18–24) had a low risk of bias. Domain-2 examined the process of identifying, retrieving, and selecting literature in each SR and showed that all (18–26) were at high risk of bias. In Domain-3, data extraction and quality evaluation, 1 SR/Meta-analyses (18) with a low risk of bias and 8 SRs/Meta-analyses (19–26) with an uncertain risk of bias were identified. Domain-4 evaluated the overall results and the combined results of each study and showed that 4 studies (19–21, 24) had a high risk of bias and 5 studies (18, 22, 23, 25, 26) had an uncertain risk of bias. Of the 9 SRs/Meta-analyses (18–26) included, all were evaluated as high risk of bias. Table 5 and Figure 4 present the ROBIS results for each SR.

**Table 5:** Risk of bias of the included systematic reviews assessed by risk of bias in systematic reviews.

First author/year	Phase 2				Phase 3
	1. Study eligibility criteria	2. Identification and selection of studies	3. Data collection and study appraisal	4. Synthesis and findings	Risk of bias in the review
Soto 2021	L	H	L	U	H
Burgstaller 2018	L	H	U	H	H
Benedetto 2011	L	H	U	H	H
Elbatarny 2020	L	H	U	H	H
Flynn 2017	L	H	U	U	H
Hu 2014	L	H	U	U	H
Mosbahi 2018	L	H	U	H	H
Salmasi 2019	H	H	U	U	H
Wu 2019	H	H	U	U	H

L, low risk; H, high risk; U, unclear risk.

## 4. Discussion

The main objective of this overview is to assess and summarize the available clinical evidence through the currently published SRs/Meta-analyses on VSRR and TRR. However, the currently available SRs/Meta-analyses were of unsatisfactory quality through our series of scale evaluations, suggesting that we need to be more cautious about further interpreting their results.

In terms of reporting quality, 1 (23) study had relatively serious information deficiencies (<15 points) and 7 (19–22, 24–26) had moderate deficiencies (≤21 points), mainly related to the evaluation of reporting bias, risk of study bias, credibility of evidence, protocol and registration, funding source, inclusion/exclusion criteria, search strategy, data extraction, and synthesis of methods and results. Of course, the above-mentioned issues are also common problems with SRs/Meta-analyses at present, especially in terms of scheme and registration, which need our attention. If we make our scheme and register it before the study, it can make us more logical on the one hand, and on the other hand, it can be used by others to search for and find the problems in time, which is a very good pre-communication process. In addition, PICO criteria were not well represented in a significant number of included studies (19, 20, 22–24, 26), and most of the included studies did not provide a complete search formula. This is a reason to suspect that the original literature is missing.

In terms of methodological quality, key items 2 and 7 in all 9 included studies had serious deficiencies. Before the implementation of the systematic review, the authors did not clearly state the study methodology for the systematic review, did not state the existence of a written protocol or guidance document, and all studies did not provide a list of excluded literature. Only three studies (18, 19, 25) considered the risk of bias in the included studies when interpreting and discussing each outcome. These results suggested that the current SR/Meta-analyses of issues related to postoperative VSRR versus TRR were largely able to follow reporting norms, but methodological quality needs to be improved, and investigators still lack attention to protocol registration, provision of search strategies, and the risk of bias in included studies.

In terms of evidence quality, the included SRs/Meta-analyses were of low/very low quality, which may be due to the following reasons. First, the methodological quality of the included cohorts was uneven and subject to large bias. Second, the outcome indicators reported in the cohort studies were not comprehensive, resulting in small sample sizes, wide confidence intervals, and imprecise results in some subgroups. Third, for publication bias reporting, some studies reported only the funnel plot, Egger's test, and Begg's test results for the primary outcome, which may have affected the credibility of other secondary outcomes.

As we know, both VSRR and TRR are different procedures for aortic root disease, and their biggest difference is whether the native aortic valve is preserved or not during the procedure. There is no denying that the VSRR is more difficult than the TRR. As mentioned in the 2022 American College of Cardiology/American Heart Association (ACC/AHA) guidelines (27), valve-preserving aortic root replacement is justified in patients undergoing aortic root replacement if the valve is suitable for repair and performed by an experienced surgeon on a multidisciplinary aortic team. In addition, the evaluation of the patient's aortic valve as well as the overall systemic condition is particularly important when performing VSRR. Through a review of the available literature, as well as our center's experience, VSRR is aggressively performed in patients with the following conditions: (i) good aortic valve pathology with high hope of preservation; (ii) young patients, especially women of reproductive age; and (iii) patients with contraindications to anticoagulation.

It is undeniable that in the secondary studies we included and in some original studies from large centers, patients who underwent VSRR had a longer time to cross-clamping and circulatory arrest than those who underwent TRR, yet the early and late mortality rates were lower in the VSRR group than those in the TRR group (28, 29). There were also very few complications in those receiving VSRR, especially the stroke rate during hospitalization, which was only half that of patients who received TRR (24). Several studies have shown a significant decrease in bleeding/embolic/endocarditis events to varying degrees as well (30–32). Beyond the above problems, the rate of valve re-intervention in patients undergoing VSRR is of real concern to everyone. Among the 5 included studies (18, 21–23, 26), only the re-intervention rate of VSRR was higher in the study by Wu et al. (26) and was approximately 4 times higher than that of patients in the TRR group. The rest of the studies did not differ from the TRR group of patients. However, this is quite acceptable given that the incidence of reintervention was only 3% higher in the VSRR group than in the TRR group in the study [VSRR: 4.9% (95% CI 0.008–0.090), TRR: 1% (95% CI 0.001–0.017)]. Therefore, we said that VSRR should be the first choice for patients if conditions permit.

## 5. Limitation

Although we have made a more detailed assessment and summary, we still cannot avoid some limitations. Firstly, multiple scales were used in the quality evaluation part of this study, and the subjectivity of the researchers in evaluating the literature could lead to bias and consequently affect the evaluation results. Secondly, SRs/Meta-analyses published more than 5.5 years were generally considered to have reduced timeliness, whereas the cycle of cardiovascular disease-related literature was even shorter (3 years) (33), and some of the studies in this review were published earlier and their contents need to be updated. Finally, there were differences in the level of centers and operators performing the VSRR procedure, and bias in the results was inherently unavoidable.

## 6. Conclusion

The current SRs/Meta-analyses point to many benefits of VSRR, including reduced early and late mortality after aortic root surgery and reduced incidence of valve-related adverse events, but the methodological quality of the relevant studies was low, and there was a lack of high-quality evidence to support them. Large-sample, multicenter clinical randomized controlled trials are necessary, and we need more rigorous and methodologically sound SRs/Meta-analyses to draw clear conclusions that can guide clinical practice.

## Data Availability

The original contributions presented in the study are included in the article/Supplementary Material, further inquiries can be directed to the corresponding authors.
